# BDNF Val^66^Met Positive Players Demonstrate Diffusion Tensor Imaging Consistent With Impaired Myelination Associated With High Levels of Soccer Heading: Indication of a Potential Gene-Environment Interaction Mechanism

**DOI:** 10.3389/fneur.2019.01297

**Published:** 2019-12-11

**Authors:** Liane E. Hunter, Yun Freudenberg-Hua, Peter Davies, Mimi Kim, Roman Fleysher, Walter F. Stewart, Richard B. Lipton, Michael L. Lipton

**Affiliations:** ^1^The Gruss Magnetic Resonance Research Center, Albert Einstein College of Medicine and Montefiore Medical Center, The Bronx, NY, United States; ^2^Division of Geriatric Psychiatry, Northwell Health, Glen Oaks, NY, United States; ^3^Litwin-Zucker Center for the Study of Alzheimer's Disease, The Feinstein Institute for Medical Research, Northwell Health, Manhasset, NY, United States; ^4^Department of Epidemiology & Population Health, Albert Einstein College of Medicine and Montefiore Medical Center, The Bronx, NY, United States; ^5^Department of Radiology, Albert Einstein College of Medicine and Montefiore Medical Center, The Bronx, NY, United States; ^6^Sutter Health Research Center, Walnut Creek, CA, United States; ^7^Department of Neurology, Albert Einstein College of Medicine and Montefiore Medical Center, The Bronx, NY, United States; ^8^Department of Psychiatry & Behavioral Sciences, Albert Einstein College of Medicine and Montefiore Medical Center, The Bronx, NY, United States; ^9^The Dominick P. Purpura Department of Neuroscience, Albert Einstein College of Medicine and Montefiore Medical Center, The Bronx, NY, United States

**Keywords:** BDNF Val^66^Met, diffusion tensor imaging, myelination and re-myelination, soccer heading, mild traumatic brain injury

## Abstract

The purpose of this study was to examine the potential effect modifying role of the BDNF Val^66^Met polymorphism on the association of soccer heading with white matter microstructure. We studied 312 players enrolled in the ongoing Einstein Soccer Study, a longitudinal study of amateur soccer player in New York City and surrounding areas. At enrollment and 2 years later, total heading in the prior 12 months (12-mo.) was estimated using an established self-report instrument and diffusion tensor imaging (DTI) was performed. Generalized Estimating Equations (GEE) logistic regression models were employed to test effect modification by the BDNF Val^66^Met polymorphism on the association between 12-mo. heading exposure and DTI. We identified a significant interaction of 12-mo heading^*^BDNF Val^66^Met genotype on the presence of low Radial Diffusivity, a DTI marker associated with myelination. Only Met (+) players demonstrated significantly reduced odds of low RD [OR (95 % CI): −2.36 (−3.53, −1.19)] associated with the highest vs. lowest quartile of 12-mo heading exposure. BDNF Val^66^Met (+) soccer players with long-term exposure to high levels of heading exhibit less low Radial Diffusivity, suggesting impaired re-myelination may be a substrate of the previously reported association between heading and poor functional outcomes in soccer players.

## Introduction

The game of soccer is a global phenomenon played by over 265 million people worldwide (1). Since the early 1980's, there has been growing awareness regarding the potential adverse neurological consequences associated with repetitive heading of the ball in soccer (2–4). As described in Maher et al.'s review article (5), the literature examining the effects of soccer heading on functional impairment is variable. While some studies have demonstrated no association between heading and poor neuropsychological performance (6, 7), a growing body of evidence suggests that heading in soccer is associated with lasting functional impairments (8–12); Given the apparent idiopathic response, it is essential to understand factors that may contribute to individual differences in vulnerability to heading, including potential genetic modifiers of outcomes.

Diffusion Tensor Imaging (DTI) is an MRI modality capable of detecting changes to the white matter microstructure following Traumatic Brain Injuries (TBIs) (13). There is now evidence to suggest that subconcussive head injury is associated with aberrations in brain tissue microstrutcture (8, 14–16). Radial Diffusivity (RD) is a DTI marker which reflects myelination wherein high RD is associated with myelin loss following injury and low RD is associated with myelination (17).

Brain Derived Neurotrophic Factor (BDNF) is a protein most well-known for facilitating neuronal maturation and synaptic plasticity (18). Recent animal studies have shown that BDNF also promotes re-myelination after a brain lesion (19, 20). The BDNFVal^66^Met polymorphism represents a substitution of valine to methionine in the BDNF pro-peptide, which decreases secretion of BDNF into the synapse (18). Recent studies have demonstrated that presence of the Met allele is associated with impaired cognition (21) and more emotional symptoms (22) in mild TBI patients.

Understanding genetic modifiers of the association of soccer heading with brain microstructure can lead to identification of high risk individuals as well as provide insight into the underlying mechanism(s) of the microstructural consequences of heading. Because no study to date has examined this role for the BDNF Val^66^Met polymorphism, the goal of the present study was to explore the effect modifying role of the Met allele on the association between long-term heading and DTI. We hypothesized that Met (+) players who head the most would demonstrate a more adverse profile on DTI outcomes, consistent with axonal injury and/or impaired re-myelination, compared to Met (–) players.

## Methods

Participants: Soccer players enrolled in the Einstein Soccer Study from November 2013 through August 2018 were included in this analysis. Recruitment and study procedures are described in detail elsewhere (9, 23, 24). In brief, adult amateur soccer players in the New York City and surrounding areas were recruited via print, internet advertisement and through soccer leagues, clubs, and colleges. A research team member contacted qualifying individuals, confirmed eligibility and willingness to participate in the full longitudinal study, and invited enrollment. Players were eligible if they were aged 18–55, played soccer for more than 5 years, were currently playing soccer more than 6 months per year, and were fluent in English. Exclusion criteria included a self-reported diagnosis of schizophrenia, bipolar disorder, a known neurologic disorder (e.g., stroke or transient ischemic attack), or illicit drug use within 30 days based on urine toxicology. At enrollment, the initial study visit participants completed: (1) Written Informed Consent, (2) A web-based demographic questionnaire (e.g., gender, race, years of education), (3) HeadCount-12m (described below), (4) Venipuncture to obtain blood for genotyping (described below), and (5) a DTI scan. At the final visit, 2 years after enrollment, subjects repeated a DTI scan. All study procedures were approved by the Institutional Review Board at the Albert Einstein College of Medicine.

Healthy subjects, that were used as a reference group for imaging analysis (see below), consisted of non-athletes ages 18–50 years old. Exclusion criteria for healthy subjects included a history of a head injury, a psychiatric illness (schizophrenia, bipolar disorder, anxiety, depression), diabetes, hypertension, heart disease, or contraindication to MRI. All soccer players and controls provided written informed consent that was approved by the Institutional Review Board at the Albert Einstein College of Medicine.

HeadCount-12mo. Questionnaire: HeadCount-12mo is a questionnaire that estimates their total number heads in the prior 12-months [refer to Levitch et al. (9) and Rubin et al. (14) for a detailed explanation of Head Count-12mo]. In brief, participants are asked a series of questions pertaining to their soccer play during practice and competition in indoor and outdoor settings: (1) the number of months per year active in each setting; (2) the average number of competitive soccer games per week; (3) the average number of headers per game; (4) the average number of practices per week; (5) the average number of headers per practice. Total heading in the past year was estimated by multiplying average headers in each setting by number of sessions per week in each setting, converted to month, and then multiplying by the number of months of play per year. Subtotals in each setting were summed to obtain an estimate of total 12-mo. heading. HeadCount-12m also asks participants to report the number of years they have been playing soccer at a similar frequency and their lifetime concussion history. Subjects were instructed to consider a concussion any head injury for which they sought or were asked to seek medical attention.

## Diffusion Tensor Imaging (DTI)

Details of image acquisition and processing procedures have been previously described (14).

In brief, imaging was performed using a 3.0T Philips Achieva TX scanner (Philips Medical Systems, Best, The Netherlands) with a 32-channel head coil. T1-weighted 3D magnetization-prepared rapid acquisition of gradient echo imaging was performed with TR/TE/TI = 9.9/4.6/900 ms, flip angle 8°, 1 mm^3^ isotropic resolution, 240 × 188 × 220 matrix. Diffusion tensor imaging was performed using 2D single-shot EPI with 32 diffusion encoding directions, b-value = 800 s/mm^2^, TR = 10 s, TE = 65 ms, 2 mm^3^ isotropic resolution, 128 × 120 matrix, 70 slices. Image processing was performed using a high-performance computing system running the Community Enterprise Operating System (CentOS) Linux distribution which incorporates the FSL package (25). The 32 diffusion-weighted image sets (32 b = 800 s/mm^2^ images) were corrected for head motion and eddy current effects by using an affine registration algorithm, with the b = 0 s/mm^2^ image as the target volume. Brain extraction was performed using Brain Extraction Tool (BET), and a white matter mask that was generated using FAST was applied to limit data analysis to white matter only. All images were reviewed by a board-certified neuroradiologist for structural abnormalities or evidence of prior trauma including hemorrhage. Trained reviewers checked the raw and processed images for image quality, artifacts and aberrant results of processing. Problematic or suboptimal images were excluded from the analysis. Diffusion parameter images for each control participant were transformed to match each soccer player using Subject-based registration (SURE-Quant), which has been shown to minimize potential registration errors (26). A voxel-wise analysis of covariance (VANCOVA) was performed to identify subject-specific abnormalities in DTI parameters including Fractional Anisotropy (FA), Radial Diffusivity (RD), Axial Diffusivity (AD), and Mean Diffusivity (MD). As previously described (26, 27), this is a method that permits us to identify clusters (e.g., a grouping of voxels) of DTI abnormalities by comparing each individual subject to a group of normative controls. In the present study, DTI parameter image volumes from each soccer player were compared to those of 110 healthy controls to identify subject-specific abnormalities in DTI parameters. To guard against Type I errors, we only retained clusters comprising at least 100 voxels that were all significant at *p* </= 0.01 (8, 26). For each subject, we extracted total volume (mm^3^) of abnormally low and abnormally high FA, RD, AD, and MD, yielding 8 summary measures from each player for subsequent statistical analysis. All imaging analyses were adjusted for effects of age and gender.

Whole blood (5 ml) was obtained via venipuncture at enrollment. The BDNF Val^66^Met polymorphism (rs6265, G > A) was genotyped using the Global Screening Array-24.v1.0 (GSA) chip and genotype data was analyzed with the Golden Helix SVS software. Data quality control (QC) was performed at both “per-marker” and “per-individual” level (28). The rs6265 variant had a call rate of 98.7% (meaning only 1.3% of the individuals had a missing genotype for this variant), which is above the > = 95% genotype call rate cutoff. The Hardy Weinberg Equilibrium (HWE) predicts the expected genotype frequencies from the observed allele frequencies in a population and genotyping errors often lead to departure from HWE (29). No significant deviation from Hardy-Weinberg Equilibrium (HWE) was observed for rs6265 (*P* = 0.68). At the “per-individual” level, QC samples with an overall genotyping failure rate of >10% on the GSA chip were removed from analysis.

## Statistical Analysis

All analyses were conducted using STATA 15.0. Baseline demographic group differences between Met (–) vs. Met (+) players were assessed using Student's *t*-test or Wilcoxon Rank sum test for continuous variables and chi-squared or Fischer's exact test for categorical variables. Our 12-months heading predictor variable demonstrated extreme positive skewness, based on visual inspection of a histogram; therefore, this variable was categorized into quartiles, yielding groups approximately equal in size. To determine if the presence of the Met allele modified the association between 12-mo. heading and presence of low or high DTI parameters, we utilized Generalized Estimating Equations (GEE) logistic regression models with independent covariance structure. GEE is an approach that models data from different visits as independent while accounting for within subject correlation (30). Gender, age, race, years of education, maximum number of alcoholic beverages consumed in a week, years playing soccer at a similar frequency and number of lifetime concussions (0,1, 2+) were considered as possible confounder. A backward-stepwise regression approach with *p* < 0.05 as criterion for retention was used to determine the final models. To correct for multiple comparisons, we applied a False Discovery Rate (FDR) correction at *p* = 0.10 (31). In the event of a significant interaction, *post-hoc* analyses stratified by Met status were conducted.

## Results

Our final sample consisted of 312 soccer players and 422 DTI scans obtained from the baseline and two-year visits. Seventy-three (73%) percent of the participants were men. The average age of players in this analysis was 25.9 (SD = 7.5). Table 1 demonstrates that Met (–) and Met (+) players differed significantly with regards to gender and therefore this variable was used as a covariate in all subsequent GEE analyses. The results of the multivariate logistic regressions estimating the odds of high FA (*p* = 0.33), low FA (*p* = 0.86), high AD (*p* = 0.16), low AD (*p* = 0.05), high MD (*p* = 0.10), low MD (*p* = 0.22), and high RD (*p* = 0.16) were not significant after FDR correction. However, there was a significant interaction (*p* = 0.02) of 12-mo. heading^*^Met allele on the odds of low RD, aka abnormality in RD compared to a group of controls, after correction for multiple comparisons. As demonstrated in Table 2, only Met (+) players demonstrated significantly reduced odds of low RD associated with the highest vs. lowest quartile of 12-mo heading exposure (Table 2).

**Table 1:** Baseline characteristics in BDNFVal^66^Met (–) vs. BDNFVal^66^Met (+) players (*N* = 312).

		**Met (–) players (*N* = 221)**	**Met (+) players (*N* = 91)**	***p***
**Variable (continuous)**				
Age, years		24 (21–29)	23 (21–27)	0.32
Education, years		15.5 (3.0)	15.8 (2.47)	0.43
Years playing at similar frequency^a^		11 (7–16)	10 (5–16)	0.50
**Variable (categorical)**				
Gender	Male	167 (76)	58 (64)	0.03
Race	White	148 (67)	66 (72)	0.34
Past/Present Smoker	Yes	67 (30)	23 (25)	0.37
Alcoholic drinks/week	0	60 (27)	17 (19)	0.07
	1–2	99 (45)	34 (37)	
	3–7	47 (21)	32 (35)	
	8–14	14 (6)	8 (9)	
	14+	1 (1)	0 (0)	
Lifetime concussion(s)^a^	0	149 (68)	48 (54)	0.053
	1	31 (14)	20 (22)	
	2+	38 (18)	21 (24)	

*Continuous variables are reported as mean (sd) for education and median (IQR) for age and years at similar frequency. Categorical variables are reported as frequency (%)*.

a *Data missing in five subjects*.

**Table 2:** Odds Ratio and 95% Confidence intervals (CI) from regression models estimating the association of 12-mo heading on the presence of low Radial Diffusivity on DTI imaging.

	**BDNF Val^66^Met stratified analyses^+^**			
	**Met (–) players^a^**		**Met (+) players^b^**	
	**OR (95% CI)**	***P***	**OR (95% CI)**	***P***
**Heading exposure** (range of heading)				
Q1 (0–174)	—	—	—	—
Q2 (178–548)	−0.00 (−0.73, 0.73)	0.99	−0.76 (−1.84, 0.32)	0.17
Q3 (556–1459)	−0.08 (−0.79, 0.63)	0.82	−0.89 (−2.09, 0.30)	0.14
Q4 (1460–22828)	−0.24 (−0.96, 0.48)	0.51	−2.36 (−3.53, −1.19)	<0.001^*^

+ P-value for Met*Heading interaction from GEE model: p = 0.02.

a *Model adjusted for gender and years of education*.

b *Model adjusted for gender*.

* *p < 0.05*.

## Discussion

To our knowledge, this study is the first to demonstrate an effect of genotype on microstructural tissue outcomes from sub-concussive heading in soccer. We have found that compared to Met (–) players, the odds of low RD associated with high levels of 12-mo. heading is reduced by 89% in players with at least one copy of the BDNF Met allele.

There is still much debate regarding the long-term consequences of exposure to repetitive soccer heading; however, understanding the distinct pathological mechanisms underlying exposures may help to elucidate the basis for heterogeneous outcomes. Prior studies combing histology and DTI have demonstrated that high RD specifically reflects myelination and lower RD is associated with myelin loss following experimental injury (32–34). Moreover, preclinical studies have demonstrated that BDNF promotes re-myelination following de-myelinating brain lesions (19, 20). One possible mechanism of this observation is that BDNF regulates autophagy (35), which in turn is essential for proper myelination (36). In the context of this evidence, our results suggest that impaired re-myelination, due to presence of the Met allele, is a potential pathological substrate of functional deficits, which are associated with high levels of sub-concussive heading (8, 9). However, it must be noted that RD is indeed a surrogate marker for myelination as there is no myelin-specific imaging measure available at present. Another potential explanation is that our findings reflect a phenomenon in which high-heading negates neuroplastic changes, such as enhanced myelination, that are conferred by physical activity and training. Finally, there is a risk of chance despite our adjustment for multiple comparisons.

Several limitations to this preliminary study must be considered. First, this a candidate gene association study that always requires replication in a different and larger sample. Number of lifetime concussions was not determined as a significant confounder in the models; however, Met (+) players demonstrated a borderline trend toward a greater number of lifetime concussions; a fact that may have contributed to our results. Likewise, we have previously reported gender differences in DTI outcomes from soccer heading (14). Though we adjusted for gender in our analyses, we were not powered to detect a heading^*^gender^*^ BDNF Val^66^Met interaction; which limits our ability to address the combined effect of the Met allele and gender on white matter abnormalities from soccer heading. Moreover, we estimate exposure level based on self-report and therefore we cannot entirely dismiss potential bias due to reporting or recall error. However, our prior validation of HeadCount, treatment of exposure as categorical variable and the consistency of HeadCount across multiple timeframes and domains of measurement make reporting error unlikely to bias our findings. The method of exposure assessment does not permit us to address the role of biomechanical features of individual impacts. Our imaging analysis utilized a voxel-wise approach, that examines the aggregate abnormalities across the whole brain (26); thus we cannot identify specific abnormal white matter tracts in soccer players. Finally, our cohort of young, healthy amateur soccer players is generally reflective of a large segment of soccer participants worldwide, but differences from other populations and age groups may limit generalization of our findings to other populations.

The maximum population benefit protecting against the potential adverse effects of soccer heading will accrue when injury can be detected prior to overt clinical dysfunction. Our preliminary findings point to impaired re-myelination, associated with the BDNF Met allele, as a pathological mechanism underpinning the adverse consequences of soccer heading. Further study of the role of BDNF in the exposure-response relationship, in both human and preclinical studies of repetitive sub-concussive head impacts are warranted and may serve as the basis for assessment of player risk and motivate development and testing of remyelination treatments.

## Data Availability Statement

This manuscript contains previously unpublished data. Request to access the dataset should be directed to ML (michael.lipton@einstein.yu.edu).

## Ethics Statement

This study involving human participants was reviewed and approved by Albert Einstein College of Medicine Institutional Review Board. The participants provided their written informed consent to participate in this study.

## Author Contributions

LH: study concept and design, analysis and interpretation of data, and critical revision of manuscript for intellectual contents. RF: acquisition, analysis and interpretation of imaging data, and critical revision of manuscript for intellectual content. YF-H, PD, MK, WS, and RL: interpretation of data and critical revision of manuscript for intellectual content. ML: study concept and design and interpretation of data, and critical revision of manuscript for intellectual content and study supervision.

### Conflict of Interest

RL was the Edwin S. Lowe Professor of Neurology at the Albert Einstein College of Medicine in New York. He receives research support from the NIH: 2PO1 AG003949 (mPI), 5U10 NS077308 (PI), R21 AG056920 (Investigator), 1RF1 AG057531 (Site PI), RF1 AG054548 (Investigator), 1RO1 AG048642 (Investigator), R56 AG057548 (Investigator), U01062370 (Investigator), RO1 AG060933 (Investigator), K23 NS09610 (Mentor), K23AG049466 (Mentor), K23 NS107643 (Mentor). He also receives support from the Migraine Research Foundation and the National Headache Foundation. He serves on the editorial board of Neurology, senior advisor to Headache, and associate editor to Cephalalgia. He has reviewed for the NIA and NINDS, holds stock options in eNeura Therapeutics and Biohaven Holdings; serves as consultant, advisory board member, or has received honoraria from: American Academy of Neurology, Alder, Allergan, American Headache Society, Amgen, Autonomic Technologies, Avanir, Biohaven, Biovision, Boston Scientific, Dr. Reddy's, Electrocore, Eli Lilly, eNeura Therapeutics, GlaxoSmithKline, Merck, Pernix, Pfizer, Supernus, Teva, Trigemina, Vector, Vedanta. He receives royalties from Wolff's Headache 7th and 8th Edition, Oxford Press University, 2009, Wiley and Informa. The remaining authors declare that the research was conducted in the absence of any commercial or financial relationships that could be construed as a potential conflict of interest.

## References

[1] Kunz M Big count 265 million playing football. In: FIFA Magazine. Federation Internationale de Football Association (FIFA) (2007). p. 10–15. 10.1080/13673882.2007.9680857

[2] Tysvaer AT Løchen EA. Soccer injuries to the brain. a neuropsychologic study of former soccer players. Am J Sports Med. (1991) 19:56–60. 10.1177/036354659101900109 2008931

[3] Spiotta AM Bartsch AJ Benzel EC. Heading in soccer: dangerous play? Neurosurgery. (2012) 70:1-11; discussion 11. 10.1227/NEU.0b013e31823021b2 21811187

[4] Tarnutzer AA Straumann D Brugger P Feddermann-Demont N. Persistent effects of playing football and associated (subconcussive) head trauma on brain structure and function: a systematic review of the literature. Br J Sports Med. (2017) 51:1592–604. 10.1136/bjsports-2016-096593 27815240

[5] Maher ME Hutchison M Cusimano M Comper P Schweizer TA. Concussions and heading in soccer: a review of the evidence of incidence, mechanisms, biomarkers and neurocognitive outcomes. Brain Inj. (2014) 28:271–85. 10.3109/02699052.2013.865269 24475745

[6] Kaminski T Wikstrom A Gutierrez G Glutting J Purposeful heading during a season does not influence cognitive function or balance in female soccer players. J Clin Exp Neuropsychol. (2007) 29:742–51. 10.1080/13825580600976911 17852597

[7] Straume-Naesheim TM Andersen TE Dvorak J Bahr R. Effects of heading exposure and previous concussions on neuropsychological performance among Norwegian elite footballers. Br J Sports Med. (2005) 39 (Suppl. 1):i70–7. 10.1136/bjsm.2005.019646 16046359 PMC1765315

[8] Lipton ML Kim N Zimmerman ME Kim M Stewart WF Branch CA. Soccer heading is associated with white matter microstructural and cognitive abnormalities. Radiology. (2013) 268:850–7. 10.1148/radiol.13130545 23757503 PMC3750422

[9] Levitch CF Zimmerman ME Lubin N Kim N Lipton RB Stewart WF. Recent and long-term soccer heading exposure is differentially associated with neuropsychological function in amateur players. J Int Neuropsychol Soc. (2017) 24:147–55. 10.1017/S1355617717000790 28829004 PMC6554717

[10] Witol AD Webbe FM. Soccer heading frequency predicts neuropsychological deficits. Arch Clin Neuropsychol. (2003) 18:397–417. 10.1016/S0887-6177(02)00151-8 14591454

[11] Webbe FM Ochs SR. Recency and frequency of soccer heading interact to decrease neurocognitive performance. Appl Neuropsychol. (2003) 10:31–41. 10.1207/S15324826AN1001_5 12734073

[12] Moore RD Lepine J Ellemberg D. The independent influence of concussive and sub-concussive impacts on soccer players' neurophysiological and neuropsychological function. Int J Psychophysiol. (2017) 112:22–30. 10.1016/j.ijpsycho.2016.11.011 27867100

[13] Hutchinson EB Schwerin SC Avram AV Juliano SL Pierpaoli C. Diffusion MRI and the detection of alterations following traumatic brain injury. J Neurosci Res. (2018) 96:612–25. 10.1002/jnr.24065 28609579 PMC5729069

[14] Rubin TG Catenaccio E Fleysher R Hunter LE Lubin N Stewart WF. MRI-defined white matter microstructural alteration associated with soccer heading is more extensive in women than men. Radiology. (2018) 289:478–86. 10.1148/radiol.2018180217 30063172 PMC6209057

[15] Koerte IK Ertl-Wagner B Reiser M Zafonte R Shenton ME. White matter integrity in the brains of professional soccer players without a symptomatic concussion. Jama. (2012) 308:1859–61. 10.1001/jama.2012.13735 23150002 PMC4103415

[16] Myer GD Barber Foss K Thomas S Galloway R DiCesare CA Dudley J. Altered brain microstructure in association with repetitive subconcussive head impacts and the potential protective effect of jugular vein compression: a longitudinal study of female soccer athletes. Br J Sports Med. (2018) 53:1539–51. 10.1136/bjsports-2018-099571 30323056

[17] Aung WY Mar S Benzinger TL. Diffusion tensor MRI as a biomarker in axonal and myelin damage. Imaging Med. (2013) 5:427–40. 10.2217/iim.13.49 24795779 PMC4004089

[18] Chen ZY Patel PD Sant G Meng CX Teng KK Hempstead BL. Variant brain-derived neurotrophic factor (BDNF) (Met66) alters the intracellular trafficking and activity-dependent secretion of wild-type BDNF in neurosecretory cells and cortical neurons. J Neurosci. (2004) 24:4401–11. 10.1523/JNEUROSCI.0348-04.2004 15128854 PMC6729450

[19] Fulmer CG VonDran MW Stillman AA Huang Y Hempstead BL Dreyfus CF. Astrocyte-derived BDNF supports myelin protein synthesis after cuprizone-induced demyelination. J Neurosci. (2014) 34:8186–96. 10.1523/JNEUROSCI.4267-13.2014 24920623 PMC4051974

[20] Tsiperson V Huang Y Bagayogo I Song Y VonDran MW DiCicco-Bloom E. Brain-derived neurotrophic factor deficiency restricts proliferation of oligodendrocyte progenitors following cuprizone-induced demyelination. ASN Neuro. (2015) 7:1759091414566878. 10.1177/1759091414566878 25586993 PMC4720179

[21] McAllister TW Tyler AL Flashman LA Rhodes CH McDonald BC Saykin AJ. Polymorphisms in the brain-derived neurotrophic factor gene influence memory and processing speed one month after brain injury. J Neurotrauma. (2012) 29:1111–8. 10.1089/neu.2011.1930 22188054 PMC3325555

[22] Wang YJ Chen KY Kuo LN Wang WC Hsu YW Wong HS. The association between BDNF Val^66^Met polymorphism and emotional symptoms after mild traumatic brain injury. BMC Med Genet. (2018) 19:13. 10.1186/s12881-017-0518-0 29357818 PMC5776765

[23] Stewart WF Kim N Ifrah C Sliwinski M Zimmerman ME Kim M. Heading frequency is more strongly related to cognitive performance than unintentional head impacts in amateur soccer players. Front Neurol. (2018) 9:240. 10.3389/fneur.2018.00240 29740384 PMC5928847

[24] Stewart WF Kim N Ifrah CS Lipton RB Bachrach TA Zimmerman ME. Symptoms from repeated intentional and unintentional head impact in soccer players. Neurology. (2017) 88:901–8. 10.1212/WNL.0000000000003657 28148633 PMC5331870

[25] Smith SM Jenkinson M Woolrich MW Beckmann CF Behrens TE Johansen-Berg H. Advances in functional and structural MR image analysis and implementation as FSL. Neuroimage. (2004) 23 (Suppl. 1):S208–19. 10.1016/j.neuroimage.2004.07.051 15501092

[26] Suri AK Fleysher R Lipton ML. Subject based registration for individualized analysis of diffusion tensor MRI. PLoS ONE. (2015) 10:e0142288. 10.1371/journal.pone.0142288 26580077 PMC4651497

[27] Strauss SB Kim N Branch CA Kahn ME Kim M Lipton RB.. Bidirectional changes in anisotropy are associated with outcomes in mild traumatic brain injury. AJNR Am J Neuroradiol. (2016) 37:1983–91. 10.3174/ajnr.A4851 27282864 PMC5148740

[28] Anderson CA Pettersson FH Clarke GM Cardon LR Morris AP Zondervan KT. Data quality control in genetic case-control association studies. Nat Protoc. (2010) 5:1564–73. 10.1038/nprot.2010.116 21085122 PMC3025522

[29] Chen B Cole JW Grond-Ginsbach C. Departure from hardy weinberg equilibrium and genotyping error. Front Genet. (2017) 8:167. 10.3389/fgene.2017.00167 29163635 PMC5671567

[30] Hardin JW Generalized Estimating Equations. Hoboken, NJ: Wiley Online Library (2005). 10.1002/0470013192.bsa250

[31] Benjamini Y Hochberg Y Controlling the false discovery rate: a practical and powerful approach to multiple testing. J R Stat Soc. (1995) 57:289–300. 10.1111/j.2517-6161.1995.tb02031.x

[32] Song SK Sun SW Ramsbottom MJ Chang C Russell J Cross AH. Dysmyelination revealed through MRI as increased radial (but Unchanged Axial) diffusion of water. NeuroImage. (2002) 17:1429–36. 10.1006/nimg.2002.1267 12414282

[33] Ou X Sun SW Liang HF Song SK Gochberg DF. The MT pool size ratio and the DTI radial diffusivity may reflect the myelination in shiverer and control mice. NMR Biomed. (2009) 22:480–7. 10.1002/nbm.1358 19123230 PMC3711249

[34] Song SK Sun SW Ju WK Lin SJ Cross AH Neufeld AH. Diffusion tensor imaging detects and differentiates axon and myelin degeneration in mouse optic nerve after retinal ischemia. Neuroimage. (2013) 20:1714–22. 10.1016/j.neuroimage.2003.07.005 14642481

[35] Nikoletopoulou V Sidiropoulou K Kallergi E Dalezios Y Tavernarakis N. Modulation of autophagy by BDNF underlies synaptic plasticity. Cell Metab. (2017) 26:230–42 e235. 10.1016/j.cmet.2017.06.005 28683289

[36] Bankston AN Forston MD Howard RM Andres KR Smith AE Ohri SS. Autophagy is essential for oligodendrocyte differentiation, survival, and proper myelination. Glia. (2019) 67:1745–59. 10.1002/glia.23646 31162728

